# A Systematic Review on the Contribution of Artificial Intelligence in the Development of Medicines for COVID-2019

**DOI:** 10.3390/jpm11090926

**Published:** 2021-09-18

**Authors:** Carla Pires

**Affiliations:** CBIOS, Escola de Ciências e Tecnologias da Saúde, Universidade Lusófona’s Research Center for Biosciences and Health Technologies, Campo Grande 376, 1749-024 Lisboa, Portugal; p5558@ulusofona.pt

**Keywords:** artificial intelligence, COVID-2019, SARS-CoV-2, drug design, medicines, repurposing of drugs, molecular docking, molecular dynamics, in silico methods, machine learning

## Abstract

Background: COVID-2019 pandemic lead to a raised interest on the development of new treatments through Artificial Intelligence (AI). Aim: to carry out a systematic review on the development of repurposed drugs against COVID-2019 through the application of AI. Methods: The Systematic Reviews and Meta-Analyses (PRISMA) checklist was applied. Keywords: [“Artificial intelligence” and (COVID or SARS) and (medicine or drug)]. Databases: PubMed^®^, DOAJ and SciELO. Cochrane Library was additionally screened to identify previous published reviews on the same topic. Results: From the 277 identified records [PubMed^®^ (n = 157); DOAJ (*n* = 119) and SciELO (*n* = 1)], 27 studies were included. Among other, the selected studies on new treatments against COVID-2019 were classified, as follows: studies with in-vitro and/or clinical data; association of known drugs; and other studies related to repurposing of drugs. Conclusion: Diverse potentially repurposed drugs against COVID-2019 were identified. The repurposed drugs were mainly from antivirals, antibiotics, anticancer, anti-inflammatory, and Angiotensin-converting enzyme 2 (ACE2) groups, although diverse other pharmacologic groups were covered. AI was a suitable tool to quickly analyze large amounts of data or to estimate drug repurposing against COVID-2019.

## 1. Introduction

On 31 December 2019, a cluster of cases of pneumonia in Wuhan, Hubei Province, China was reported with the identification of a likely new form of coronavirus. The genetic sequence of the virus SARS-CoV-2 (severe acute respiratory syndrome coronavirus 2), which is the etiologic agent of COVID-2019 (coronavirus disease 2019) was shared by China on 12 January 2020. The first COVID-2019 case was reported outside China in Thailand on 13 January 2020, which was followed by an alarming spread of the number of cases in different regions/countries. On 11 March 2020, the COVID-2019 pandemic was declared by the World Health Organization (WHO) [1,2]. Globally, 188,655,968 confirmed cases of COVID-19, including 4,067,517 deaths have been reported to WHO on 16 July 2021, with 3,402,275,866 vaccine administered doses on 15 July 2021 [3].

SARS-CoV-2 infects the epithelial cells or immune cells, producing tissue damage. Epithelial cells and immune cells release inflammatory cytokines, such as IL-1, IL-6, IL-12, and TNFα. Inflammatory cytokines recruit innate immune cells (e.g., monocytes, macrophages, neutrophils, DCs, and NK cells) and activate adaptive immune cells (CD4+ T cells and CD8+ T cells). These occurrences may induce myelopoiesis and emergency granulopoiesis, associated with the production of sustained and excessive circulating cytokines, which may lead to a serious epithelial damage. Immunopathologic events may contribute to explain COVID-19 pathogenesis, such as lymphopenia, neutrophilia, dysregulation of monocytes and macrophages, reduced/delayed response of type I interferon (IFN-I), antibody-dependent enhancement, and cytokine storm [4]. The knowledge of COVID-2019 immunopathology may help in the comprehension and development of new treatments and/or drug repurposing (i.e., the application of old drugs to treat new diseases) [5,6,7,8]. Additionally, the measurement of serum levels of pro-inflammatory cytokines (e.g., IL-6) may be applied in the management of COVID-2019 (adult and pediatric patients), such as in the assessment of risk, monitoring of disease progression, determination of prognosis, selection of therapy. and prediction of response to treatment [7].

Because of the pandemic, the research on the development of new therapies, treatments, and vaccines against COVID-2019, including drug repurposing assumed an unprecedent significance at a global level [5]. Drug repurposing present some advantages over the discovery of new molecules/active substances, such as the reduction of production costs, the achievement of likely safe medicines, and potentially shorter development timelines [5,6]. 

The development of new treatments against COVID-2019 keep on a hot topic due to the likely risk of new virus variants, which may compromise the efficacy of treatments [9]. By definition, a “variant has one or more mutations that differentiate it from other variants in circulation” [10]. These variants may comprise the efficacy of approved treatments for COVID-2019, such as vaccines and/or other medicines. Additionally, the number of therapeutics against COVID-2019 remains limited [9,11]. 

On 18 July 2021, the only centralized authorized antiviral by the European Medicine Agency (EMA) to the treatment of COVID-2019 was remdesivir (*nucleoside analogue used to treat RNA virus infections*; DrugBank), while baricitinib is the only drug with a marketing authorization application submitted to EMA for the treatment of this disease. Baricitinib is a *janus kinase inhibitor* (JAK) *used to treat moderate to severe rheumatoid arthritis that has responded poorly to at least one TNF antagonist* (DrugBank) [11,12,13,14]. JAK inhibitors (e.g., baricitinib and ruxolitinib) are indicated in the treatment of inflammatory diseases. JAK present key pharmacological proprieties, such as convenient oral administration, favorable pharmacokinetic profile, and dual anti-inflammatory and anti-viral effects, with potential benefit in the treatment of COVID-2019 [15].

Additionally, a group of drugs is currently under rolling review for a possible use in the treatment of COVID-2019: Bamlanivimab (*neutralizing human IgG1κ monoclonal antibody against the SARS-CoV-2 spike (S) protein for use in patients aged 12 and over at high risk of developing severe COVID-19*; DrugBank) and etesevimab (*a fully human and recombinant monoclonal antibody that targets the SARS-CoV-2 surface spike protein receptor binding domain*; DrugBank), Regdanvimab (monoclonal antibody), REGN-COV2 (casirivimab/imdevimab) (*monoclonal antibody cocktail used to treat mild to moderate COVID-19*; DrugBank), and Sotrovimab (*a monoclonal antibody for the treatment of mild-to-moderate COVID-19 in patients at increased risk for death or hospitalization*; DrugBank). In addition, 4 vaccines have received a conditional marketing authorization in European Union: two COVID-19 mRNA vaccine (nucleoside-modified) (Comirnaty^®^ and Spikevax^®^); one adenovirus type 26 encoding the SARS-CoV-2 spike glycoprotein (Ad26.COV2-S^®^) (COVID-19 Vaccine Janssen), and one ChAdOx1-SARS-CoV-2 vaccine (Vaxzevria^®^) [11,12,13,14].

Recently, WHO announced the COVID-2019 solidarity therapeutic trial, which enrolls 14,200 hospitalized patients randomized, 600 hospitals and 2000 researchers from 52 countries. This trial will evaluate the safety and efficacy of three repurposing drugs against COVID-2019, as follows: artesunate (*a derivative of artemisinin, an antimalarial drug extracted from the herb Artemisia annua*), imatinib (*a small molecule tyrosine kinase inhibitor, formulated as an oral chemotherapy drug used to treat certain types of cancer*), and infliximab (*a TNFα inhibitor, a chimeric monoclonal antibody that recognizes human TNF alpha*) [16].

The knowledge of SARS-CoV-2 genome is fundamental for drug development, such as antivirals and vaccines. The genome of SARS-CoV-2 is composed of 16 nonstructural proteins (nsps) such as main protease (Mpro), papain-like protease, RNA-dependent RNA polymerase (RdRp), helicase, 4 structural proteins (envelope, membrane, spike, and nucleocapsid), and other proteins. For instance, the spike glycoprotein is involved in the interactions between the virus and receptors of host cell, while nsps proteins are especially relevant during the life cycle of the virus e.g., in the production of subgenomic RNAs. These proteins (nonstructural and structural), potentially constitute promising drug targets for the design and development of antiviral agents against COVID-19 [5,17,18].

Synthetic and natural drug candidates against the target protein of SARS-CoV-2 may be identified through the application of computational methods (e.g., molecular docking and molecular dynamics) plus artificial intelligence methodologies (AI) [19]. Molecular docking is a methodology applied to study the conformation and orientation (“pose”) of molecules into the binding site of a macromolecular target. Poses are ranked by scoring functions, which are based on searching algorithms [20]. Molecular docking is based on the static picture of the drug-target complexes in comparison with molecular dynamics methodologies that introduce flexibility to explain the drug binding process. Some of the disadvantages of molecular docking is their computational cost and the vast chemical space that is required. These disadvantages may be minimized through the application of AI methodologies, with the application of Monte Carlo tree search algorithms and/or multitask neural networks [21]. 

Molecular dynamics simulations were initially used to simulate several hundreds of atoms. However, they are currently used to simulate systems with biological relevance (e.g., proteins in solution with the explicit representation of proteins, membrane embedded proteins, or large macromolecular complexes) [22,23]. Both molecular docking and pharmacophore modelling are classified as in silico methods. Pharmacophores are applied to represent and identify molecules trough a 2D or 3D representation, with the schematic depiction of the key elements of molecular recognition. Often, pharmacophore models are used to check simple geometric and chemical functionality requirements, prior to the application of more complex approaches, such as molecular docking [24,25]. 

Additionally, AI and machine learning methods have been extensively applied in the discover of new treatments, such as vaccines, and drug repurposing for COVID-2019, as well as, in the analyze and explanation of pharmaceutical-related big data, since AI implies the simplification, acceleration and reduction the costs of drug discovering [5,19]. For instance, MolAICal software is a freely available tool (https://molaical.github.io, accessed on 7 September 2021). This tool produces 3D drug design in the 3D pocket of protein targets by AI (a deep learning model) and classical algorithm. MolAICal is composed of by two modules. The first module uses “the genetic algorithm, deep learning model trained by FDA-approved drug fragments and Vinardo score fitting on the basis of PDBbind database for drug design”. The second module applies “deep learning generative model trained by drug-like molecules of ZINC database and molecular docking invoked by Autodock Vina automatically”. The non-membrane protein SARS-CoV-2 main protease and membrane protein glucagon receptor were selected as the exploratory drug targets. Molecular docking was applied to estimate the affinities between generated molecules and protein. MolAICal could generate novel ligands with good binding scores and appropriate XLOGP (a program for the prediction of the octanol/water partition coefficients of organic compounds) values [26].

Current evidence points that AI may be used to identify diverse potential drug targets, novel and/or repurposing of drugs and vaccine candidates for the potential treatment of COVID-2019 [15]. Thus, study aim was to carry out a systematic review on the identification of repurposing drugs against COVID-2019 through the application of AI methodologies.

## 2. Materials and Methods

A systematic review on AI and the development of repurposing drugs against COVID-2019 was carried out. The Systematic Reviews and Meta-Analyses (PRISMA) checklist and flow diagram for new systematic reviews were applied [27].

### 2.1. Research Question

What are the repurposing drugs against COVID-2019 identified through AI?

### 2.2. Searched Period

The present systematic review was conducted without time restrictions during June and July 2021. PubMed^®^, SciELO, and DOAJ were browsed on 10 June 2021. Cochrane Library was browsed on 12 July 2021. All outputs were collected and stored in printed pdf files.

### 2.3. Keywords

The selected keywords were as follows: [“Artificial intelligence” and (COVID or SARS) and (medicine or drug)]. These keywords were conveniently selected to cover the boarder number of related papers.

These searches were respectively carried out in abstract and title in PubMed^®^ and Cochrane Library and in all fields in DOAJ and SciELO. The option “all fields” is not predicted in Cochrane Library. The option “abstract and title” was activated in PubMed^®^ to increase data accuracy.

### 2.4. Inclusion and Exclusion Criteria

#### 2.4.1. Inclusion Criteria

All peer-reviewed studies using AI methodologies to identify repurposed drugs against COVID-2019. Only, synthetic molecules and natural therapies were included.

#### 2.4.2. Exclusion Criteria

Studies on other topics, review studies and non-peer-reviewed studies were excluded. Repurposed candidates based on proteins and/or vaccines were excluded.

### 2.5. Searched Databases and Period

The selected databases (PubMed^®^, SciELO, DOAJ, and Cochrane Library) were screened without time limitations. These databases were conveniently selected because they contain an expressive number of peer reviewed papers and/or publications following editorial quality control: PubMed^®^ (more than 32 million citations for biomedical literature), SciELO (440,302 documents), DOAJ (6,301,494 articles records), and Cochrane Library (8630 Cochrane Reviews) on 12 July 2021. Particularly, Cochrane Library was screened aiming at identifying previous published Cochrane reviews on the same topic. 

### 2.6. Identification of Previous Systematic Reviews on Related Topics

PubMed^®^, SciELO, DOAJ and Cochrane Library were browsed on 11 July 2021, with the keywords “artificial intelligence” and “medicine or drug” in title or abstract without time limitations and with the option “systematic review” activated (if applicable, e.g., the activation of the option “systematic review” in PubMed^®^). 

Overall, 72 reviews were identified in PubMed^®^, 13 in DOAJ, 0 SciELO, and 0 Cochrane Library, with the identification of 2 reviews on related topics (one in PubMed^®^ and another in DOAJ, respectively) [5,19].

#### 2.6.1. Findings of the Review of Kaushal et al. (2020) 

In a search carried out between December 2019 and May 2020 in 5 databases, AI was applied to a rapid identification of diverse potential drug targets, novel and/or repurposing of drugs and vaccine candidates for the potential treatment of COVID-2019. The main top-ranked promising candidate drug(s)/molecule(s) are presented in Appendix A [19].

#### 2.6.2. Findings of the Review of Gurung et al. (2021) 

Two computer-aided drug designs (CADD) were reviewed: the ligand-based (when the three-dimensional structure of the target receptor is not available, e.g., AI and quantitative structure-activity relationships, QSARs) and the structured-based drug discovery (the evaluation of the binding site cavity with the three-dimensional structure of the therapeutic target proteins). These methodologies were relevant for the discovery of new or repurposing drugs against COVID-2019. 

Particularly, five groups of repurposing drugs against COVID-2019 were identified as the most relevant. These groups/drugs were, as follows: RNA polymerase inhibitors (Remdesivir; Favipiravir; Oseltamivir; Galidesivir; Sofosbuvir); 3C-like protease inhibitors (Lopinavir; Ritonavir; Ivermectin); papain-like protease inhibitor (Dissulfiram); TMPRSS2 inhibitors (Camostat mesylate; Nafamostat; Bromhexine; Enzalutamide) and inhibitors of endosomal acidification (Chloroquine and Hydroxychloroquine) [5]. 

## 3. Results

### 3.1. PRISMA Flow Diagram

**Figure 1:**
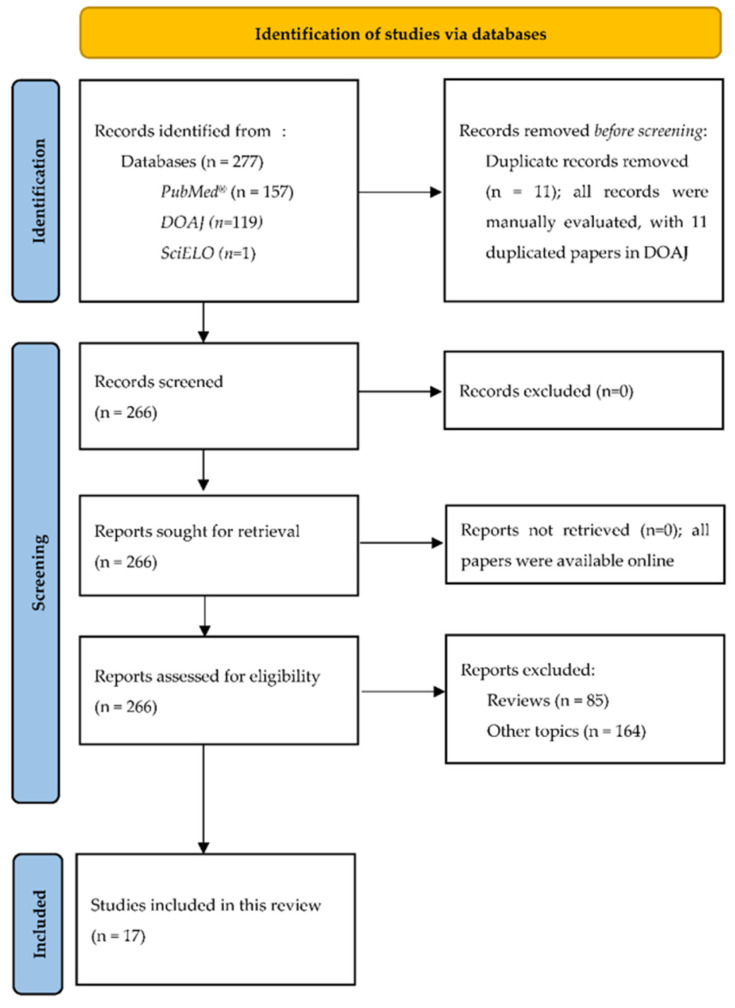
The Systematic Reviews and Meta-Analyses (PRISMA) flow diagram: selection of studies on the identification of repurposing drugs against COVID-2019 through AI [27].

The Systematic Reviews and Meta-Analyses (PRISMA) flow diagram is presented in Figure 1 [27].

### 3.2. Main Findings of the Selected Studies

Some aspects of the 17 selected studies are briefly summarized in Table 1, such as the list of key repurposed drug(s) with potential therapeutic use against COVID-2019. 

These studies are briefly described in three sections: studies with confirmatory in-vitro data (Section 3.2.1); studies with confirmatory in-vitro and/or clinical data (Section 3.2.2) and repurposing of drugs against COVID-2019 (Section 3.2.3).

#### 3.2.1. Studies with Confirmatory In-Vitro Data

Bedaquiline (used to treat tuberculosis), brequinar (immunosuppressant), celecoxib (nonsteroidal anti-inflammatory drug), clofazimine (bactericidal effect on Mycobacterium leprae), conivaptan (nonpeptide, dual antagonist of arginine vasopressin; treatment of euvolemic or hypervolemic hyponatremia), gemcitabine (antineoplastic anti-metabolite), tolcapone (catechol-O-methyltransferase inhibitor used in the therapy of Parkinson disease), and vismodegib (selectively binds to and inhibits the transmembrane protein Smoothened homologue; antineoplastic activity) were identified through AI learning and prediction processes. These 8 predicted drugs were tested for activities against a feline coronavirus in an in vitro cell-based assay. The antiviral effects were measured in feline infectious peritonitis (FIP) virus-infected feline catus whole fetus-4 (Fcwf-4) cells. All predicted drugs exhibited in vitro activities against the proliferation of a FIP virus in Fcwf-4 cells for verification of antiviral activity. Two learning database collections were applied in AI searching: one from approved drugs for SARSCoV-2, HIV, influenza, and others and other from 3CL protease inhibitors. The AI search was based on the characteristics of three types of molecular descriptors, i.e., drugs with certain molecular characteristics were preferably selected [28].

Overall, 6340 drugs were screened for their expected efficacy against SARS-CoV-2 through algorithms based on artificial intelligence, network diffusion, and network proximity. The predicted candidates were compared to 918 compounds that had been experimentally screened for their efficacy against SARS-CoV-2 in VeroE6 cells, kidney epithelial cells derived from African green monkey. VeroE6 cells were preincubated with the drugs (8 μM–8 nM), which were challenged with wild-type SARS-CoV-2 strain USA-WA1/2020. Additionally, the list of drugs in clinical trials with potential COVID-19 efficacy was screened. Overall, 13 candidates registered positive outcomes in VeroE6 cells, which were tested in human cells to confirm their clinical relevance (Huh7 cells, in a nine-point dilution series from 25 μM–100 nM). Among the most promising candidates, were Auranofin (*antirheumatic used to treat active, progressive, or destructive forms of inflammatory arthritis*; DrugBank), azelastine (*histamine H1-receptor antagonist used intranasally to treat allergic and vasomotor rhinitis and in an ophthalmic solution to treat allergic conjunctivitis*; DrugBank), digoxin (*cardiac glycoside used in the treatment of mild to moderate heart failure and for ventricular response rate control in chronic atrial fibrillation*; DrugBank), and vinblastine (*vinca alkaloid used to treat breast cancer, testicular cancer, neuroblastoma, Hodgkin’s and non-Hodgkins lymphoma, mycosis fungoides, histiocytosis, and Kaposi’s sarcoma*; DrugBank) presented a very strong anti–SARS-CoV-2 response in human cells; fluvastatin (*an HMG-CoA reductase inhibitor used to lower lipid levels and reduce the risk of cardiovascular disease including myocardial infarction and stroke*; DrugBank) presented a weaker response; and methotrexate was effective, but only at the highest concentration [29].

An AI-based platform was applied to identify combination therapies that optimally inhibited the infection of the A549 lung cell line by vesicular stomatitis virus (efficacy) while maximizing A549 viability. This technology may be applied to address a broad spectrum of infectious diseases, including the appearance of new strains e.g., COVID-2019. For instance, Amantadine (*used to treat dyskinesia in Parkinson’s patients receiving levodopa, as well as extrapyramidal side effects of medications*; DrugBank); Azithromycin (*macrolide antibiotic used to treat a variety of bacterial infections*; DrugBank); Chloroquine (antimalarial drug, see Appendix A); Omeprazole Sodium (*a proton pump inhibitor used to treat GERD associated conditions such as heartburn and gastric acid hypersecretion, and to promote healing of tissue damage and ulcers caused by gastric acid and H. pylori infection*; DrugBank) and/or Ribavirin (*a guanosine nucleoside used to treat some forms of Hepatitis C*; DrugBank) in combination with other candidates were among the AI-optimized regimes. Optimally and sub-optimally dosed combinations, registered a sevenfold difference in efficacy, which clearly proves the critical relevance of ideal drug and dose selection/identification [30].

IDentif.AI is a platform that combines AI, digital drug development and in-vitro experimentation (SARS-CoV-2 in vitro, cellular infectious disease model) to detect drug interactions and optimize the design of associations therapies with suitable doses. Viral inhibition and cell cytotoxicity of drug monotherapies and drug combinations were carried out with Vero E6 cells (African green monkey kidney). The Vero E6 cells (2 × 10^4^ cells/well) and media with and without SARS-CoV-2 treatment (100 TCID50) were added to the plates containing the drugs and the controls. The viral infection model was based on virus’s cytopathic effect (CPE). The Viral ToxGlo assay quantifies viral-induced CPEs in host cells by using cellular ATP as a marker: the decrease in cellular ATP detected is proportional to the number of viable host cells in culture, i.e., it is possible to correlate viral CPE with viral burden. The optimized combination of remdesivir (nucleoside analog), ritonavir (HIV protease inhibitor), and lopinavir (HIV-1 protease inhibitor) was identified as potentially clinically relevant. In experimental studies, a 6.5-fold enhanced efficacy in comparison to remdesivir alone. In opposition, the combination of hydroxychloroquine (antimalarial) and azithromycin (macrolide antibiotic) were relatively ineffective in vitro at clinically relevant doses [31]. For more details on the pharmacologic activity of remdesivir, ritonavir, lopinavir, hydroxychloroquine and azithromycin see Appendix A [19].

#### 3.2.2. Studies with Confirmatory In-Vitro and/or Clinical Data

The oral Janus kinase (JAK)1/JAK2 inhibitor baricitinib, which is currently used for the treatment of rheumatoid arthritis (RA). Baricitinib was proposed by an AI algorithm due to their anti-cytokine effects and as an inhibitor of host cell viral propagation (proven anti-inflammatory effects and predicted antiviral effects) [32,33]. The aberrant activation of the JAK-STAT (signal transducer and activator of transcription) signaling pathway is related to the regulation of the immune system response. The aberrant activation of the JAK-STAT occurs in certain diseases, such as RA or psoriatic arthritis. Diverse cytokines are involved in the activation of JAK/STAT, such as pro-inflammatory cytokines (e.g., IL6, IL12, IL23 or TNF). Cytokine binding to JAK/STAT activate the signaling cascade, which leads to the activation of JAKs. This mechanism may explain the potential benefit of using JAK inhibitors, such as baricitinib in the treatment of COVI-2019, regarding cytokines may be increased (e.g., cytokine storm) in COVID-2019 [7,8,15].

The AI platform (BenevolentAI) predicted that baricitinib might also inhibit Numb-associated kinases (NAKs), which are important for viral entry into a cell (i.e., prediction of antiviral effects). Particularly, inhibition of NAKs led to reduced viral infectivity with baricitinib using human primary liver spheroids. In vitro pharmacology studies of baricitinib across relevant leukocyte subpopulations and in vivo pharmacokinetics data demonstrated the inhibition of signaling of cytokines implicated in COVID-19 infection. The pharmacologic effect of barictinib is supported by both in-vitro (dual antiviral and anti-inflammatory activities in liver organoids; human liver spheroids infected with SARSCoV-2) and clinical data (4 hospitalized patients). 

Additionally, clinical, and radiologic recovery, a quick diminution in SARS-CoV-2 viral load, inflammatory markers reduced (e.g., C-reactive protein or interleukin-6), and IL-6 levels were achieved in the treatment of 4 hospitalized patients with COVID-2019. Compassionate use was authorized, regarding the previous approved application in RA [32,33].

#### 3.2.3. Repurposing of Drugs against COVID-2019

This section was created, regarding the lack of confirmatory in/vitro and/or clinical data/findings in the studies here described. The repurposed compounds were selected aiming at targeting the main coronavirus protease (6LU7). From the 31 selected repurposed compounds, remdesivir (a nucleoside analog, see Appendix A), valrubicin (*an anthracycline used intravesically in the treatment of BCG-resistant bladder carcinoma*; DrugBank), aprepitant (*a substance P/neurokinin 1 receptor antagonist used to treat nausea and vomiting caused by chemotherapy and surgery*; DrugBank), and fulvestrant (*an estrogen receptor antagonist used to treat HR+ breast cancer that may also be HER2-*; DrugBank) were identified as potential COVID-19 protease inhibitors. Additionally, a new compound ‘nCorv-EMBS’ was identified, among the best shape-based compounds. Shape-based molecules starting from the 3D shape to the pharmacophoric features of their seed compound were identified through a machine learning approach. Ligand-Receptor Docking was performed with Optimized Potential for Liquid Simulations (OPLS) algorithms to identify high affinity compounds. The selected drug candidates were subjected to Molecular Dynamic Simulations followed by absorption, distribution, metabolism, and excretion-toxicity in pharmacokinetics (ADMET) studies (AdmetSAR and Swiss ADME software explained various ADMET properties) and other analyses [34].

Kim et al. (2020) applied two computational approaches to identify existing drugs (FDA approved drugs) to prevent or reduce clinical infection of severe acute respiratory syndrome coronavirus 2 (SARS-CoV-2), as follows: (1) an AI-based binding affinity prediction platform was used to identify drugs that could block coronaviruses from entering cells by binding to angiotensin converting enzyme or transmembrane Serine Protease 2 (TMPRSS2) (first group) and (2) Disease Cancelling Technology (DCT) platform was applied to identify drugs that could attenuate the gene expression patterns induced by coronaviruses (second group). Among the drugs identified were a beta-lactam antibiotic (piperacillin sodium) (antibiotic), two antiviral agents (fosamprenavir and emricasan) and glutathione (a tripeptide for nutritional supplementation) for the first group (see Appendix A), and Vitamin E (*an antioxidant vitamin used in many skin creams and multivitamin preparations*; DrugBank), ruxolitinib (*a kinase inhibitor used to treat various types of myelofibrosis, polycythemia vera in patients who have not responded to or cannot tolerate hydroxyurea, and to treat graft-versus-host disease in cases that are refractory to steroid treatment*; DrugBank), and glutamine (an amino acid, with possible applications in parenteral nutrition; see Appendix A) for the second group [19,35].

AI and pattern recognition techniques were used for initial screening of FDA approved pharmaceuticals and nutraceuticals to target the CoV envelope (E) protein (a membrane protein, which is implicated in assembly and release of the virus inside the host). Molecular docking simulations were performed between the ligands and target protein to screen 9 ligand molecules: nafcillin (*a penicillin derivative antibiotic used to treat susceptible staphylococcal infections*; DrugBank); nabumetone (*an NSAID used to treat osteoarthritis and rheumatoid arthritis*; DrugBank); octacosanol (*1-octacosanol is a straight-chain aliphatic 28-carbon primary fatty alcohol, with cholesterol-lowering effects, antiaggregatory properties, cytoprotective use, and ergogenic properties*; DrugBank); cinametic acid (monocarboxylic acid); lauric acid; ascorbyl palmitrate (anti-oxidant); palmidrol (*a cannabinoid receptor-inactive eCB-related molecule used as prophylactic in helping to prevent respiratory viral infection*; DrugBank); salmeterol (*a long-acting beta-2 adrenergic receptor agonist used to treat asthma and COPD*; DrugBank) and guaifenesin (*an expectorant commonly found in OTC products for the symptomatic relief from congested chests and coughs associated with cold, bronchitis, and/or other breathing illnesses*; DrugBank) [36].

Diverse computational methods were used to predict the repurposed drugs: machine learning techniques namely Support Vector Machine, Random Forest, k-Nearest Neighbour, Artificial Neural Network, and Deep Learning. The drugs of ‘DrugRepV’ repository, with anticorona activity experimentally validated (IC50/EC50) were extracted. Prediction models with Pearson’s were constructed. After scanning the DrugBank, these models were used to predict repurposed drug candidates against coronaviruses. The top ranked molecules were verteporfin (*a benzoporphyrin derivative used to treat pathological myopia, ocular histoplasmosis, and choroidal neovascularization in macular degeneration*; DrugBank), alatrofloxacin (*a fluoroquinolone antibiotic used to treat a variety of bacterial infections*; DrugBank), metergoline (*an ergot-derivative that acts as an antagonist at certain 5-HT receptor subtypes and as an agonist at dopamine receptors*; DrugBank), rescinnamine (*an angiotensin-converting enzyme inhibitor used as an antihypertensive drug*; DrugBank), leuprolide (*a peptide-based GnRH receptor superagonist used for the palliative treatment of prostate cancer, uterine leiomyomata, endometriosis, and central precocious puberty*; DrugBank), and telotristat ethyl (*a tryptophan hydroxylase inhibitor*; DrugBank) with high binding affinity. Finally, molecular docking strategies against the spike protein complex with ACE receptor were used to validate the top rank molecules [37].

Human host dependency genes were evaluated as potential drug targets for antiviral diseases. Particularly, these genes were indispensable for the successful viral infection. Database retrieval, literature mining and de novo prediction using artificial intelligence-based algorithms were used to interrogate extensive drug-target interactions. From 2352 FDA approved drugs, diverse potential antiviral drug candidates were identified for the treatment of coronaviruses (e.g., SARS-CoV-2), flaviviruses (e.g., Zika virus) and influenza viruses. Among the most promising drug candidates against Coronaviridae viruses in the group of the top ten repurposed FDA-approved drugs against Coronaviridae viruses were Fostamatinib (*a spleen tyrosine kinase inhibitor used to treat chronic immune thrombocytopenia after attempting one other treatment*; DrugBank), Baricitinib (a janus kinase inhibitor, see Appendix A) and Simvastatin (to treat hypercholesterolemia) according to the Joint P-score and Baricitinib (Rheumatoid arthritis), Lusutrombopag (to treat thrombocytopenia; *an orally bioavailable thrombopoietin receptor (TPOR) agonist*; DrugBank) and Bivalirudin (to treat thrombocytopenia; *a synthetic 20 residue peptide (thrombin inhibitor) which reversibly inhibits thrombin*, Drug-Bank) according Joint PN-score [38].

An estimation model was developed through the complex heterogeneous biomedical knowledge graph, SemNet aiming at predicting missing links in biomedical literature regarding drug repurposing. Among the most ranked drug classes were anti-inflammatory, nucleoside analogues, protease inhibitors, antimalarials, envelope proteins, and glycoproteins. For instance, highly ranked predicted links to SARS-CoV-2 were identified for human leukocyte interferon, recombinant interferon-gamma (*a Type 1 inflammatory cytokine; antitumor properties, antiviral activities, and important immunoregulatory functions*; DrugBank), cyclosporine (*a steroid-sparing immunosuppressant used in organ and bone marrow transplants as well as inflammatory conditions such as ulcerative colitis, rheumatoid arthritis, and atopic dermatitis*; DrugBank), antiviral therapy, zidovudine (*a dideoxynucleoside used in the treatment of HIV infection*; DrugBank), chloroquine (antimalarial drug, see Appendix A), methotrexate (*an antineoplastic agent used the treatment of a wide variety of cancers as well as severe psoriasis, severe rheumatoid arthritis, and juvenile rheumatoid arthritis*; DrugBank), artemisinin (*treatment of malaria*; DrugBank), alkaloids, glycyrrhizic acid (it is extracted from the root of the licorice plant; Glycyrrhiza glabra; *hepatoprotective drug in cases of chronic hepatitis*; DrugBank), quinine (*an alkaloid derived from the bark of the cinchona tree; it is used as an antimalarial drug*; DrugBank), flavonoids, amprenavir (*a protease inhibitor used to treat HIV infection*; DrugBank), suramin (*a polyanionic compound used in the treatment of African trypanosomiasis, with potent antineoplastic properties;* DrugBank), complement system proteins, fluoroquinolones, albuterol (*a beta-2 adrenergic receptor agonist used to treat asthma, bronchitis, COPD, as well as prevent exercise induced bronchospasms*; DrugBank), ciprofloxacin (*a second generation fluoroquinolone used to treat various susceptible bacterial infections*; DrugBank), quinolone antibacterial agents, and hydroxymethylglutaryl-CoA reductase inhibitors [39].

Knowing that the combination of angiotensin-converting enzyme inhibitors and calcium channel blockers is used for pulmonary hypertension in critical settings and that some of these drugs independently were therapeutically relevant in COVID-2019, the AI plataform BIOiSIM was used to conducted a in silico modelling, with some of these drugs. This platform used know preclinical in vitro and in vivo datasets to simulate systemic therapy and site-of-action penetration of the calcium channel blockers and angiotensin-converting enzyme compounds to tissues implicated in the pathogenesis of COVID19. Among the identified drugs were spirapril, lisinopril, and captopril (angiotensin-converting enzyme inhibitors) and lacidipine and verapamil (calcium channel blockers). For instance, spirapril (*an ACE inhibitor antihypertensive drug used to treat hypertension*; DrugBank), lisinopril (*an ACE inhibitor used to treat hypertension, heart failure, and acute myocardial infarction*; DrugBank), and captopril (*an ACE inhibitor used for the management of essential or renovascular hypertension, congestive heart failure, left ventricular dysfunction following myocardial infarction, and nephropathy*; DrugBank) seems to be suitable candidates as repurposing drugs against COVID-2019 [40].

A workflow of combined in silico methods using the supercomputer MOGON and chemical libraries consisting of FDA approved drugs were applied to identify novel drug candidates against COVID-19, namely virtual screening (obtained by AutoDock VINA), molecular docking (obtained by AutoDock 4.2.6) findings and ROC probability indicated as the most ten ranked compounds binding to spike protein:

- Simeprevir (*direct-acting antiviral agent that inhibits HCV NS3/4A protease to treat chronic hepatitis C virus (HCV) infection in adults with HCV genotype 1 or 4*; DrugBank),
- Paritaprevir (*a direct acting antiviral agent used in combination with other antiviral agents for the treatment of Hepatitis C Virus (HCV) infections*; DrugBank),
- Velpatasvir (*a NS5A inhibitor used to treat chronic hepatitis C infections in patients without cirrhosis or with compensated cirrhosis*; DrugBank),
- Rifapentine (*an antibiotic agent used in the treatment of pulmonary tuberculosis*; DrugBank),
- Eribulin (*a microtubule inhibitor used to treat metastatic breast cancer and metastatic or unresectable liposarcoma*; DrugBank),
- Teniposide (*a cytotoxic drug used as an adjunct for chemotherapy induction in the treatment of refractory childhood acute lymphoblastic leukemia*; DrugBank),
- Trabectedin (*an alkylating agent approved for the treatment of unresectable or metastatic soft tissue sarcoma (liposarcoma or leiomyosarcoma)*; DrugBank),
- Ivermectin (*an anti-parasite medication used to treat head lice, onchocerciasis, strongyloidiasis, ascariasis, trichuriasis, and enterobiasis*; DrugBank),
- Ledipasvir (*a direct-acting antiviral agent used to treat specific hepatitis C virus (HCV) infections in combination with other antiviral agents*; DrugBank), and
- Nystatin (*a polyene ionophore antifungal used to treat cutaneous, mucocutaneous, and gastrointestinal mycotic infections, particularly those caused by Candida species*; DrugBank).

Similar methodologies were applied to find the 10 compounds most likely to bind to nucleocapsid protein:

- Paritaprevir, trypan blue (*a dye used as a visualizing aid to stain the epiretinal membranes during ophthalmic surgical vitrectomy procedures, thereby facilitating removal of the tissue*; DrugBank),
- Simeprevir,
- Dihydroergotamine (*an ergot alkaloid used in the acute treatment of migraine headache and cluster headache*; DrugBank),
- Conivaptan (*an antidiuretic hormone inhibitor used to raise serum sodium levels*; DrugBank),
- Ergotamine (*an alpha-1 selective adrenergic agonist vasoconstrictor used to treat migraines with or without aura and cluster headaches*; DrugBank),
- Venetoclax (*a BCL-2 inhibitor used to treat chronic lymphocytic leukemia, small lymphocytic lymphoma, or acute myeloid leukemia*; DrugBank),
- Idarubicin (*an anthracycline antineoplastic agent used to treat acute myeloid leukemia (AML) in adults*; DrugBank),
- Ivermectin and
- Nystatin.

Finally, the same methodologies were applied to find the 10 most likely compounds that bind to 2′-o-ribosemethyltransferase:

- Conivaptan,
- Lifitegrast (*a medication used to treat dry eye disease*; DrugBank),
- Dihydroergotamine, Ergotamine,
- Eltrombopag (*a thrombopoietin receptor agonist used to treat thrombocytopenia or aplastic anemia associated with various etiologies*; DrugBank),
- Ponatinib (*a kinase inhibitor used to treat patients with various types of chronic myeloid leukemia (CML)*; DrugBank),
- Lumacaftor (*a protein chaperone used in combination with ivacaftor for the treatment of cystic fibrosis in patients who are homozygous for the F508del mutation in the CFTR gene*; DrugBank),
- Nilotinib (*a kinase inhibitor used for the chronic phase treatment of Chronic Myeloid Leukemia (CML) that is Philadelphia chromosome positive and for the treatment of CML that is resistant to therapy containing imatinib*; DrugBank),
- Regorafenib (*a kinase inhibitor used to treat patients with metastatic colorectal cancer, unresectable, locally advanced, or metastatic gastrointestinal stromal tumors, and hepatocellular carcinoma*; DrugBank), and
- Aprepitant (*a substance P/neurokinin 1 receptor antagonist used to treat nausea and vomiting caused by chemotherapy and surger*; DrugBank) [41].

##### 3.2.3.1. Repurposing of Drugs against COVID-2019: Association of Drugs

The association of pirfenidone (*an agent used for the treatment of idiopathic pulmonary fibrosis (IPF)*; DrugBank) and melatonin (*an endogenous hormone produced by the pineal gland that regulates sleep-wake cycles and when provided exogenously has beneficial effects on sleep-onset latency; available as an over-the-counter supplement*; DrugBank) was identified as a candidate treatment to reduce the virus infection in non-severe symptomatic patients through combined system biology and artificial intelligence-based approaches. An artificial neural network (ANN) was applied to identify a potential relationship between the nodes of a network (i.e., proteins) with a certain phenotype—Therapeutic Performance Mapping System (TPMS). This methodology (TPMS-ANN) intends to integrate the known information about the functional interaction of proteins with patient public available data. Drug targets and their respective applications were collected from DrugBank. The molecular description of the indications was based on a collection of associations between biological processes and molecular effectors (defined as BED, Biological Effectors Database, from Anaxomics Biotech) (Company’s Locations: Laussane; Barcelona; and United Kingdom). Pirfenidone and melatonin in combination strongly modulated the cytokine storm subset [42].

Abdulla et al. (2020) also reported an AI-based platform to identify optimal doses and combination therapies through the massively screening of 12 drug/dose parameter space. For instance, to treat a broad spectrum of infectious disease (e.g., COVID-2019) [30]. Remdesivir, Ritonavir and Lopinavir were successfully tested together (see studies with confirmatory in-vitro data) [31].

##### 3.2.3.2. Repurposing of Drugs: Alternative Therapies

An ontology-based side-effect prediction framework (OSPF) and Artificial Neural Network (ANN)-based deep learning was used to evaluate the Traditional Chinese Medicine prescriptions. These prescriptions were officially recommended in China for the treatment of COVID-19 (official recommended list), but side effects may be expected. The safest identified medicines were as follows: Qingfei Paidu Decoction (QFPD-T), Huashi Baidu Formula (HSBD-F), Zhongqifangzi (PMSP), GCT-CJ, Shenfu zhusheye (SF-ZSY), and Hanshiyufen fang (HSYF-F). Importantly, these medicines were suggested as supplementary treatment for COVID-19 [43].

Natural compounds from Traditional Chinese Medicine herbs were also analyzed, with the consultation of the Traditional Chinese Medicine Systems Pharmacology Database (TCMSP). These repurposed natural compounds were classified according to scores (DeepCPI P-score and DeepCPI PN-score. Among the top ten repurposed natural compounds against Coronaviridae viruses were Lysergol (Pharbitidis Semen), Atropine (Lycii Cortex, Hyoscyami Semen) and Solanocapsine (Solanum Nigrum) according to DeepCPI P-score were and Solanocapsine (Solanum Nigrum), Vitexifolin C (Viticis Fructus) and Dehydroeffusal (Junci Medulla) according to DeepCPI PN-score [38].

Virtual screening (obtained by AutoDock VINA), molecular docking (obtained by AutoDock 4.2.6) results and ROC probability were used to identify the top 10 natural compounds from literature binding to spike protein: Euphol, Loniflavone, Amyrin, Procyanidin, Crinine, Quercetin, IlexsaponinB2, Strictinin, Quercetin-3-o-rutinoside, and Punicalagin. The same methodologies were applied to identify the 10 most likely natural compounds from literature likely to bind to nucleocapsid protein, as follows: IlexsaponinB1, IlexsaponinB2, Procyanidin, Crinine, Strictinin, IlexsaponinB3, Rutin, Forsythiaside, Punicalagin and TirucallinA, as well as, to identify the 10 most likely natural compounds to bind to 2′-o-ribosemethyltransferase, as follows: Loniflavone, Friedelin, TingeninB, Hoslunddiol, Wogonoside, Procyanidin, Baicalin, IlexsaponinB2, Punicalagin and TirucallinA. In this study, natural compounds from the ZINC database were also screened. The strongest interactions were respectively found for ZINC252515584 (1R,3S,6S,7E,13S,16R,17R,21S,22S)-28-Hydroxy-17-[(2R,4R,5S,6R)-4-hydroxy-5-[(2S,4R,5R,6R)-5-hydroxy-4-(2-methoxy-6-methylbenzoyl)oxy-6-methyloxan-2-yl]oxy-6-methyloxan-2-yl]oxy-3,22-dimethyl-23,26-dioxo-24,27-dioxapentacyclo [23.2.1.01,6.013,22.016,21]octacosa-4,7,14,25 (28)-tetraene-4-carboxylic acid) (spike protein); ZINC27215482 ((1R,4S,7S)-4-benzyl-9-[(1R,4S,7S)-4-benzyl-3,6-dioxo-2,5,16-triazatetracyclo [7.7.0.02,^7^.01^0^,1^5^]hexadeca-10,12,14-trien-9-yl]-2,5,16-triazatetracyclo [7.7.0.02,^7^.01^0^,1^5^]hexadeca-10 (15),11,13-triene-3,6-dione) (nucleocapsid protein), and ZINC15675938 ((6S)–N-(4-chlorophenyl)-6-[3-(naphthalen-2-yl)-1,2,4-oxadiazol-5-yl]-1H,4H,5H,6H,7H-imidazo [4,5-c]pyridine-5-carboxamide) (2′-o-ribose methyltransferase) [41].

A supercomputer-driven pipeline for in silico drug discovery using enhanced sampling molecular dynamics, with temperature replica exchange enhanced sampling and ensemble docking was applied. Supercomputing docking was run, with billion-plus compound screens. Massively parallel supercomputing was used to quickly sample the configurational space of protein drug targets. Diverse model systems were simulated, such as S (Spike) Protein Receptor Binding Domain (RBD)/“Apo” (PDB:6W41) or S Protein RBD/Complexed with ACE2 (PDB:6W41). Authors did not divulge all findings, but Quercetin (*a natural flavonoid found in foods and natural supplement products*; DrugBank) and Hypericin (*Hypericin is under investigation for the treatment of Cutaneous T-cell Lymphoma*; DrugBank) were among the compounds identified in the top 1% preliminary results [44].

## 4. Discussion

Overall, the number of selected studies was limited (*n* = 17), although an expressive number of potential repurposed drugs against COVID-2019 has been identified. The 17 selected studies were distributed, as follows: studies with confirmatory in-vitro data and/or clinical data (*n* = 6); repurposing of drugs against COVID-2019 (*n* = 8), including alternative therapies (*n* = 2), and association of repurposed drugs (*n* = 1) (Table 1). 

In general, research was based on international collaborations, which seems to confirm the high complexity of the research on the present topic. The selected studies were mainly from USA (*n* = 10; 6 out of 10 international collaborations) and China (*n* = 5; all international collaborations), which are the countries with more publications at a global level [45]. USA and China were followed by Singapore or India (*n* = 3) and Italy or Germany (*n* = 2). The remaining countries only participated in just one study (Taiwan, Turkey, Hungary, UK, Sweden, Saudi Arabia, Spain, and Australia).

Generally, the AI methodologies were scarcely described in the selected studies; the methods of the selected studies not seemed to be fully reproductible; and in vitro or clinical findings were only reported in a limited number of studies. For instance, diverse AI platforms (e.g., IDentif.AI, BenevolentAI, or BIOiSIM) were described (Table 1), although few information on these platforms were provided in the selected studies [28,30,31,33,35,40,42]. As expected, machine learning approaches, AI deep learning, AI-based algorithms, molecular docking, and/or in silico methodologies were commonly reported [34,36,37,38,41,44], regarding they are frequently used in drug discovering. Some studies reported the application of previous developed AI methodologies, which may have facilitated study implementation. However, the sensitivity, specificity, or predictive value of the developed models and/or specific details on the application of these methodologies were generally limited or not reported in the selected studies.

### 4.1. Studies with Confirmatory In-Vitro and/or Clinical Data

Only, six studies reported in-vitro and/or clinical data to evaluate the potentially application of the identified drugs in the treatment of COVID-2019 [28,29,30,31,32,33]. Positively, all studies exhibited in vitro and/or clinical activity. These findings support the use of AI to identify drug candidates for the treatment of COVID-2019. In-vitro, in-vivo and/or clinical studies are highly recommended and necessary for the validation of safety and efficacy of potentially new medicines for the treatment of COVID-2019. However, these methodologies are highly expensive, time consuming, and requiring the involvement of experts.

Importantly, some of these drug candidates are currently enrolled in clinical trials (individually or in association with other drugs). All information about the clinical trials here reported may be consulted at ClinicalTrials.gov: https://clinicaltrials.gov/ (accessed on 17 September 2021). For instance, brequinar (e.g., NCT04575038), celecoxib (e.g., NCT04488081), clofazimine (e.g., NCT04465695), amantadine (e.g., NCT04952519), azithromycin (e.g., NCT04329832), chloroquine (e.g., NCT04443270), omeprazole (e.g., NCT04834752), ribavirin (e.g., NCT04828564), remdesivir (e.g., NCT04431453), ritonavir (e.g., NCT04960202), lopinavir (e.g., NCT04372628), hydroxychloroquine (e.g., NCT04340544), baricitinib (e.g., NCT04421027). However, these trials are currently under implementation and/or results are not yet published. In opposition, clinical trials for bedaquiline, conivaptan, gemcitabine, tolcapone, vismodegib, auranofin, azelastine, digoxin, vinblastine and fluvastatin related to the treatment of COVID-2019 were not identified in this database on 20 July 2021.

Some drug candidates, such as auranofin, azelastine, digoxin, vinblastine, fluvastatin, amantadine, omeprazole and/or ribavirin were not previously reported in the review of Kaushal et al. (2020), which proves the relevant potential AI in the identification of new molecules with potential application in treatment of COVID-2019 [19,29].

### 4.2. Repurposing of Drugs for COVID-2019, Including Alternative Therapies

Some of the identified repurposed drugs were also reported in the review of Kaushal et al. (2020), such as remdesivir, piperacillin sodium, fosamprenavir, emricasan, glutathione, glutamine, baricitinib, chloroquine, and amprenavir. Among the most prevalent pharmacologic groups of the repurposed drugs were antivirals, antibiotics, anti-inflammatories, antineoplastics, and/or ACE2, which is in line with the findings of Kaushal et al. (2020) [19]. However, repurposed drugs from diverse other pharmacologic groups were also reports.

Overall, diverse new repurposed drugs against the treatment of COVID-2019 were suggested in this systematic review through AI techniques [34,35,36,37,38,39,40,41], such as:

- valrubicin,
- aprepitant,
- dihydroergotamine, *
- bivalirudin,
- eltrombopag,
- eribulin,
- fulvestrant,
- idarubicin,
- ivermectin,
- ledipasvir,
- lifitegrast,
- nystatin,
- regorafenib,
- trypan blue,
- vitamin E,
- ruxolitinib,
- nafcillin,
- nabumetone,
- octacosanol,
- cinametic acid,
- trabectedin,
- lauric acid,
- acorbyl palmitrate,
- palmidrol,
- salmeterol,
- simvastatin,
- guaifenesin,
- verteporfin,
- metergoline,
- rescinnamine,
- leuprolide,
- lusutrombopag,
- telotristat,
- fostamatinib,
- tofacitinib,
- etoricoxib,
- ziprasidone,
- interferon-gamma;
- cyclosporine,
- zidovudine,
- methotrexate,
- artemisinin,
- glycyrrhizin acid,
- quinine,
- suramin,
- albuterol,
- ciprofloxacin,
- spirapril,
- lisinopril, and
- captopril.

These repurposed drugs were not previously described in Kaushal et al. (2020), but some of these candidates are currently under clinical trials (ClinicalTrials.gov on 15 August 2021), such as: aprepitant (e.g., NCT04470622); bivalirudin (e.g., NCT04366921), eltrombopag (e.g., NCT04516837); ivermectin (e.g., NCT04646109), ledipasvir (e.g., NCT04530422), simvastatin (e.g., NCT04348695); vitamin E (e.g., NCT04570254); ruxolitinib (e.g., NCT04362137); palmidrol (e.g., NCT04568876); fostamatinib (e.g., NCT04579393); tofacitinib (e.g., NCT04415151); cyclosporine (e.g., NCT04392531); methotrexate (e.g., NCT04610567); artemisinin (e.g., NCT04387240); glycyrrhizin acid (e.g., NCT04487964); quinine (e.g., NCT04553705); albuterol (e.g., NCT04681079); ciprofloxacin (e.g., NCT04748120); lisinopril (e.g., NCT04467931) and captopril (e.g., NCT04355429). 

In general, the reported clinical trials are still being runed, with a restricted number of clinical trials with published results in ClinicalTrials.gov. Impressively, from the 3670 clinical trials related to COVID-2019 and registered in the database ClinicalTrials.gov, only 80 had published results on 17 September 2020 (https://clinicaltrials.gov/ct2/results?cond=covid&age_v=&gndr=&type=Intr&rslt=With&Search=Apply (accessed on 17 September 2021) [18]. It is likely that some of the repurposed drugs and new molecules will be approved to be used in the treatment of COVID-2019 in the next year. Unfortunately, diverse molecules did not demonstrate positive clinical outcomes in clinical trials on the use of some candidates to treat COVID-2019 such as remdesivir, hydroxychloroquine, lopinavir, and interferon regimens that presented little or no effect on hospitalized patients with COVID-19 (NCT04315948) [46].

It seems, further in vivo and/or clinical evaluations of some candidates are required. For instance, the number of reported studies with in-vitro and/or clinical evaluation was scarce in the present review (see Section 3.2.1, Section 3.2.2 and Section 4.1). Clinical trials are essential to provide adequate evidence to ensure the safety and effectiveness of medicines against COVID-19 [47].

#### 4.2.1. Drug Repurposing: Combination of Drugs

Associations of drugs were identified as potentially useful in the treatment of COVID-2019 or in the management of their complications [30,31,42]. Drug associations may be clinically relevant if they increase the treatment efficacy of COVI-2019, regarding the number approved treatments for COVID-2019 is still limited [11]. 

The identified associations of (1) pirfenidone plus melatonin to reduce viral infection (non-severe symptomatic patients); (2) Amantadine; Azithromycin; Chloroquine; Omeprazole Sodium and/or Ribavirin or (3) Lopinavir, Remdesivir, and Ritonavir were not previously suggested in the systematic review of Kaushal et al. (2020) [19]. These associations were also not reported in the database ClinicalTrials.gov on 21 July 2021 [30,31,42]. Additionally, other combinations of candidates are reported to treat COVID-2029, such as Lopinavir-Ritonavir (a combination of two HIV protease inhibitors) or baricitinib plus remdesivir [15,47]. Ritonavir enhances the pharmacokinetic/pharmacodynamics of Lopinavir (boosting efficacy) [47]. The association of baricitinib and remdesivir improved time to recovery with fewer serious adverse events (e.g., thrombosis), but with transaminase increase that must be supervised [15].

Clearly, AI may play a central role in the discover of new associations of drugs as well as the corresponding dosages for the treatment of COVID-2019 [30,31,40,42]. 

#### 4.2.2. Drug Repurposing: Natural Therapies

Regarding natural therapies, only a limited number of candidates was identified, such as in Traditional Chinese Medicine prescriptions [38,41,43,44]. However, diverse combined in silico methods (virtual drug screening, molecular docking, and supervised machine learning algorithms) were applied to identify new drugs for the treatment of COVID-2019. Medicinal plants with potential antiviral activity against SARS-CoV-2 were described in a recent review of Rehman, et al. (2021), as follows: *Allium sativum*, *Mentha piperita*, *Lianhua qing wen*, and *Zingiber officinale (ginger)*, *Syzygium aromatic (Clove)*, *Cassia fistula*, *Lagenaria breviflorus*, *Phyllanthus amarus*, and *Citrullus colocynthis*. All reported plants presented proved efficacy against respiratory viral infections, but without available data/evidence in the treatment of COVID-2019 [47].

FDA-approved medicines and natural compound datasets from literature mining were screened for drug repurposing. Candidates interacting with SARS-CoV-2 target proteins (spike protein, nucleocapsid protein, and 2′-o-ribose methyltransferase) were selected with ZINC database (39,442 compounds) (https://zinc.docking.org/, accessed on 17 September 2021), which was supported by the super-computer MOGON. Interactions with the spike protein were strongest for simeprevir (*a direct-acting antiviral agent that inhibits HCV NS3/4A protease to treat chronic hepatitis C virus (HCV) infection in adults with HCV genotype 1 or 4*; DrugBank), euphol (tetracyclic triterpene alcohol; the main constituent of the sap of the medicinal plant Euphorbia tirucalli, with anti-cancer activity) and ZINC252515584 [41,48]. The strongest interactions were as follows: simprevir, euphol and ZINC252515584, with the spike protein; paritaprevir (*a direct acting antiviral agent used in combination with other antiviral agents for the treatment of Hepatitis C Virus (HCV) infections*; DrugBank), Ilexsaponin B1 and ZINC27215482, with the nucleocapsid protein, and conivaptan (*an antidiuretic hormone inhibitor used to raise serum sodium levels*; DrugBank), loniflavone and ZINC15675938, with the 2′-o-ribose methyltrans-ferase. For instance, conivaptan, paritaprevir, simeprevir, loniflavone, and procyanidin were identified as potential dual inhibitors [41].

### 4.3. Future Research

Further studies are required to check potential application of these candidates in the treatment of COVID-2019 (natural or synthetic compound), such and controlled clinical trials in different populations. Studies applying AI to develop new molecules/treatments or associations of drug candidates against COVID-2019 seems to be lacking. For instance, repurposing of natural candidates against COVID-2019 is likely to be undervalued, regarding there are a significant number of bioactive compounds [49]. The application of AI to monitor the health status of severe and moderate COVID-2019 patients is recommended, such as during clinical trials or in observational studies (e.g., post-approval of medicines).

AI may have other relevant roles in clinical settings, such as in the titration of some medicines against COVID-2019, such as steroids. For instance, computed tomography images were evaluated with an AI tool to titrate steroids in the treatment of COVID-19 pneumonia. A tailored treatment was achieved for 3 patients in Renmin Hospital, Wuhan University, China. These patients, who were initially unresponsive to treatment of steroids, fully recovered after tailoring steroids therapy through AI [50]. This type of evaluations through the application of images may be applied to other types of diagnostic methods e.g., to check the accuracy of diagnosis or the success of chirurgic procedures.

Besides the application of AI in the development of drug repurposing, AI may be successfully used in COVID-2019 detection, diagnosis, screening, classification, or prediction, with significantly better outcomes, scale-up, timely responses, and sometimes outperforming humans in healthcare tasks [51]. Thus, future use of AI in COVID-2019 research may go beyond drug repurposing or other related therapeutic topics, such as dose adjustments, discover of new molecules and/or associations/combinations of repurposed candidates.

### 4.4. Study Limitations

Considering that only one author participated in collection and analyze of studies, selection bias may have been introduced. Aiming at minimizing this potential bias, all findings were registered and double checked. Positively, no conflict regarding the inclusion or exclusion of studies was found. A limited number of databases was considered, although they comprise a significant number of peer reviewed articles. 

## 5. Conclusions

AI showed to be an efficient tool to quickly analyze large amounts of data, to estimate drug repurposing against COVID-2019, to identify association of these repurposed drugs or to estimate dosage adjustments. AI methodologies were developed. These methodologies seem to be relevant in diverse settings, such as to develop new drugs, drug repurposing, quick analysis of information on COVID-2019 or other topics. For instance, AI algorithms, platforms and/or machine learning techniques were applied to predict the effect of known therapies (repurposing of drugs) to treat COVID-2019. In general, AI methodologies were limited described in the selected studies. Thus, it may be difficult to reproduce some studies and/or experimental settings 

Diverse repurposing drugs with potential application in the treatment of COVID-2019 were identified (e.g., know synthetic drugs or natural compounds). Repurposed drugs were mainly from antivirals, antibiotics, anticancer, anti-inflammatory and ACE2 groups, although drugs from diverse other pharmacologic groups were identified as potentially relevant. The number of reported in-vitro and/or clinical studies was limited, although the majority of identified drugs are undergoing official clinical trials according to the public information of ClinicalTrials.gov. Thus, the approval of more treatments enrolling repurposed drugs in the following years is expectable.

Interestingly, many of the identified drug candidates are in clinical trials according to the registers of public database: ClinicalTrials.gov (https://clinicaltrials.gov/ (accessed on 17 September 2021). However, almost all reported clinical trials still don’t have published results. At least one tyrosine kinase inhibitor (fostamatinib) was identified as potential drug candidate against COVID-2019 in the present systematic review. For instance, the tyrosine kinase inhibitor imatinib will be tested as a repurposed drug in the WHO COVID-19 Solidarity Therapeutics Trial, which seems to support the potential relevance of tyrosine kinase inhibitor as repurposed drugs against COVID-2019 [16,38]. 

In comparison to the findings of the review of Kaushal et al. (2020) (a previous review on a similar topic) diverse repurposing drugs are reported in the present review (please see Section 4.2), which confirm the relevance of AI in this field [19].

## Figures and Tables

**Table 1:** Some relevant aspects of the 17 selected studies.

Author(s); Year Reference	Country(ies) of Origin	Key Repurposed Drug(s) Potentially Relevant against the Treatment of COVID-2019	Key Used AI Methodologies
(Ke, Peng, Yeh, et al., 2020) [28]	Taiwan	Bedaquiline Brequinar Celecoxib Clofazimine Conivaptan Gemcitabine Tolcapone Vismodegib	First, an an AI platform was defined to identify potential old/repurposed drugs with anti-coronavirus activities (or potential anti-coronavirus activity). Second, AI predicted drugs were tested for their activity against a feline coronavirus in vitro. Third, results of assays were introduced in the AI system. A Deep Neural Network algorithm was used to identify the most relevant descriptors, with the generation of different weightings to generate AI prediction models.
(Morselli, do Valle, Zitnik, et al., 2021) [29]	USA, Turkey, Hungary	Auranofin Azelastine Fluvastatin Methotrexate Vinblastine	AI based on algorithms, network diffusion, and network proximity, tasking to rank 6,340 drugs, regarding their potential efficacy against SARS-CoV-2. Multimodal technology was required to fuse the prediction of all algorithms, since the predictive algorithm did not offer consistently reliable outcomes. The top-ranked drugs were screened in human cells.
(Abdulla, Wang, Qian, et al., 2020) [30] *	China, Singapore	Amantadine Azithromycin Chloroquine Omeprazole Sodium (optional) Ribavirin (optional) - These repurposing drugs were evaluated in combination.	An AI-based platform was used to interrogate 12 drug/dose parameters space. Combination therapies that optimally inhibit A549 lung cell infection by vesicular stomatitis virus within 3 days of project start were identified. This AI project utilized a quadratic relationship between drug/dose inputs and efficacy/safety outputs to successfully identify the drug-dose parameter space.
(Blasiak, Lim, Seah, et al., 2020) [31] *	China, Singapore, USA	Lopinavir Remdesivir Ritonavir - These repurposing drugs were evaluated in combination.	A platform (IDentif.AI) that pairs experimental validation with AI and digital drug development was used. Workflow of the project IDentif.AI: 1) clinically relevant concentrations based on and dose–response curves and maximal plasma concentration; 2) in vitro testing of combination therapies; combination therapies were determined through an orthogonal array composite (OACD) design; 3) IDentif.AI analysis: drug–drug interactions and clinically relevant drug-dosage combinations; and 4) biological validation.
Studies with confirmatory in-vitro and/or clinical data (Section 3.2.2)			
(Schultz, Vera, Sinclair et al., 2020) [32]	USA	Baricitinib	
(Stebbing, Krishnan, de Bono et al., 2020) [33]	UK, USA, Italy, Sweden, Singapore	Baricitinib	BenevolentAI (an artificial AI platform) identified baricitinib as a potential COVID-19 drugs. Details/information on BenevolentAI works were limited.
Repurposing of drugs against COVID-2019 (Section 3.2.3)			
(Nayarisseri, Khandelwal, Madhavi et al., 2020) [34]	India, Saudi Arabia	Aprepitant Fulvestrant Remdesivir Valrubicin	A machine learning approach was employed. Particularly, repurposed drugs were selected based on their capacity of targeting the main coronavirus protease (6LU7) using ligand-receptor Docking (optimized potential for liquid simulations algorithms to identify high affinity compounds). Additionally, candidates were subjected to Molecular Dynamic Simulations followed by ADMET (absorption, distribution, metabolism, excretion, and toxicity) studies.
(Kim, Zhang, Cha et al., 2020) [35]	USA	Emricasan Fosamprenavir Glutamine Glutathione Piperacillin sodium Ruxolitinib Vitamin E	Two computational approaches were applied. Fist, a high-throughput AI-based binding affinity prediction platform was used to identify FDA approved drugs with potential capacity to block coronaviruses from entering cells by binding to ACE2 (angiotensin-converting enzyme) or TMPRSS2 (Transmembrane Serine Protease 2). Second, the Disease Cancelling Technology (DCT) platform was used to identify FDA approved drugs, which may attenuate the gene expression patterns induced by coronaviruses.
(Das G., Das, T., Chowdhury et al., 2021) [36]	India	Ascorbyl palmitrate Cinametic acid Guaifenesin Lauric acid Nabumetone Nafcillin Octacosanol Palmidrol Salmeterol	AI deep learning techniques, in silico methodologies) and pattern recognition techniques were used to screen FDA approved pharmaceuticals and nutraceuticals to target CoV envelope (E) protein. A protein involved in the assembly and release of the virus inside the host. Multiple opensource drug databases were considered, such as ChEMBL v.26, Enamine Bio reference Compounds (https://www.enaminestore.com/products/bioreference-compounds, accessed on 17 September 2021) and Chemoinformatic tools and database (https://chemoinfo.ipmc.cnrs.fr/TMP/tmp.32396/e-Drug3D_1930_v3.sdf, accessed on 17 September 2021).
(Rajput, Thakur, Mukhopadhyay et al., 2021) [37]	India	Alatrofloxacin Metergoline Rescinnamine Rescinnamine Telotristat ethyl Verteporfin	Robust computational methods using machine learning techniques, such as Support Vector Machine, Random Forest, k-Nearest Neighbour, Artificial Neural Network, and Deep Learning were developed by the authors to predict the repurposed drugs.
(Li, Yao, Cheng et al., 2021) [38]	China, USA	Baricitinib Bivalirudin Fostamatinib Lusutrombopag Simvastatin	1) Public genetic screening data were successively interrogated to identify human-specific host dependency genes, i.e., indispensable genes for effective viral infections. 2) Extensive drug-target interactions were interrogated through diverse methodologies, such as database retrieval, literature mining and de novo prediction using AI-based algorithms.
(McCoy, Gudapati, He, Horlander, 2021) [39]	USA	Amprenavir Albuterol Artemisinin Chloroquine Ciprofloxacin Cyclosporine Fluoroquinolones Hydroxymethylglutaryl-CoA reductase inhibitors Methotrexate Quinolone antibacterial agents Suramin Zidovudine	A link prediction model was developed (an AI text mining model). The biomedical knowledge graph, SemNe was used to predict missing links in biomedical literature, regarding drug repurposing. TransE, CompleX, and RotatE based methods were used to visualize knowledge graph embeddings and link prediction results using in a web application.
(Chakravarty, Antontsev, Khotimchenko et al., 2021) [40]	USA	Captopril Lisinopril Spirapril	The plataform BIOiSIM (an AI-integrated mechanistic modeling platform) was used to simulate systemic therapy of Calcium Channel Blockers (CCBs) and ACE compounds in tissues related to the COVID-19 pathogenesis, namely the disposition and site-of-action penetration (in silico modeling). *BIOiSIM is a* *dynamic, biology-driven platform that provides a scalable computational prediction of in vivo pharmacokinetic-pharmacodynamic (PK-PD) phenomena.*
(Kadioglu, Saeed & Efferth, 2021) [41]	Germany	Conivaptan Dihydroergotamine Eltrombopag Ergotamine Eribulin Idarubicin Ivermectin Ledipasvir Lifitegrast Lumacaftor Nilotinib Nystatin Paritaprevir Ponatinib Regorafenib Rifapentine Simeprevir Teniposide Trabectedin Trypan blue Velpatasvir Venetoclax	Diverse combined in silico methods (virtual drug screening, molecular docking, and supervised machine learning algorithms) were used in a workflow to identify repurposed drug against COVID-19.
Repurposing of drugs against COVID-2019: association of drugs (Section 3.2.3.1)			
(Artigas, Coma, Matos-Filipe et al., 2020) [42]	Spain	Pirfenidone plus melatonin	The mechanism of action of pirfenidone and melatonin was simulated by using the previously described Therapeutic Performance Mapping System (TPMS) technology (an AI-based approach). GUILDify v2.0 web server was used to confirm the effect of pirfenidone and melatonin against SARS-CoV-2 infection. This web server is able to calculate the neighbourhoods of the human biological network related to the host-key points (e.g., for SARS-CoV infection) and simultaneously affected by specific drugs.
Note: References [30,31] are also related to the combination of repurposing medicines.			
Repurposing of drugs: alternative therapies (Section 3.2.3.2)			
(Wang, Li; Song, et al., 2021) [43]	China, Australia	GCT-CJ Hanshiyufen fang (HSYF-F) Huashi Baidu Formula (HSBD-F) Qingfei Paidu Decoction (QFPD-T) Shenfu zhusheye (SF-ZSY) Zhongqifangzi (PMSP) Recommended as supplementary treatment against COVID-2019.	An ontology-based side-effect prediction framework (OSPF) was developed based on a previous work and Artificial Neural Network (ANN)-based deep learning. The Traditional Chinese Medicine prescriptions for the treatment of COVID-19 (officially recommended in China) were evaluated.
(Li, Yao, Cheng et al., 2021) [38]	China, USA	Atropine (Lycii Cortex, Hyoscyami Semen) Dehydroeffusal (Junci Medulla) Lysergol (Pharbitidis Semen) Solanocapsine (Solanum Nigrum) Solanocapsine (Solanum Nigrum) Vitexifolin C (Viticis Fructus)	1) Public genetic screening data were successively interrogated to identify human-specific host dependency genes, i.e., indispensable genes for effective viral infections. 2) Extensive drug-target interactions were interrogated through diverse methodologies, such as database retrieval, literature mining and de novo prediction using AI-based algorithms.
(Kadioglu, Saeed & Efferth, 2021) [41]	Germany	Amyrin Baicalin Crinine Euphol Forsythiaside Friedelin Hoslunddiol IlexsaponinB1 IlexsaponinB2 IlexsaponinB3 Loniflavone Procyanidin Punicalagin Quercetin Quercetin-3-o-rutinoside Rutin Strictinin TirucallinA TingeninB Wogonoside ZINC252515584 ZINC27215482 ZINC15675938	Diverse combined in silico methods (virtual drug screening, molecular docking, and supervised machine learning algorithms) were used in a workflow to identify repurposed drug against COVID-19.
(Acharya, Agarwal, Baker et al., 2020) [44]	USA, Italy	Hypericin Quercetin	An enhanced sampling molecular dynamics (MD) and ensemble docking was used supercomputer-driven pipeline for in silico drug discovery.

* Studies also reported in the section on repurposing of drugs against COVID-2019: association of drugs (Section 3.2.3.1).

## Data Availability

Not applicable.

## Appendix A

**Table A1:** Top-ranked promising candidate drug(s)/molecule(s) COVID-2019 that were identified through AI in Kaushal et al. (2020) [19].

Drug Candidates	DrugBank Summary (Summary and/or Indication) [12,13]	ATC Classification [14]	Drug Candidates	DrugBank Summary (Summary and/or Indication) [12,13]	ATC Classification [14]
Abacavir	*Antiviral nucleoside reverse transcriptase inhibitor used in combination with other antiretrovirals for the treatment of HIV.*	J05AF Nucleoside and nucleotide reverse transcriptase inhibitors	Ixazomib	*A monoclonal antibody used with other medications to treat multiple myeloma in patients who have received one other therapy already.*	L01XG Proteasome inhibitors
Adenosine	*Medication used in myocardial perfusion scintigraphy and to treat supraventricular tachycardia.*	C01EB Other cardiac preparations	Lopinavir	*An HIV-1 protease inhibitor used in combination with ritonavir to treat human immunodeficiency virus (HIV) infection.*	J05AR Antivirals for treatment of HIV infections, combinations.
Afatinib	*Antineoplastic agent used for the treatment of locally advanced or metastatic non-small cell lung cancer (NSCLC) with non-resistant EGFR mutations or resistance to platinum-based chemotherapy.*	L01EB Epidermal growth factor receptor (EGFR) tyrosine kinase inhibitors	Lumacaftor	*A protein chaperone used in combination with ivacaftor for the treatment of cystic fibrosis in patients who are homozygous for the F508del mutation in the CFTR gene.*	R07AX Other respiratory system products
Almitrine mesylate	*Almitrine is a respiratory stimulant that enhances respiration by acting as an agonist of peripheral chemoreceptors located on the carotid bodies. It is used in the treatment of chronic obstructive pulmonary disease. It is also reported to have a potentially beneficial effect in treating the noctural oxygen desaturation without impairing the quality of sleep.*	R07AB Respiratory stimulants	Mannitol	*A sugar alcohol used to test for asthma, to reduce intracranial and intraocular pressure, to measure glomerular filtration rate, and to manage pulmonary symptoms associated with cystic fibrosis.*	A06AD Osmotically acting laxatives
Amprenavir	*Protease inhibitor used to treat HIV infection.*	J05AE Protease inhibitors	Meglumine	*An amino sugar found in iodinated contrast media.*	P01CB Antimony compounds
Asunaprevir	*A potent hepatitis C virus (HCV) NS3 protease inhibitor. It has been shown to have a very high efficacy in dual-combination regimens with daclatasvir in patients chronically infected with HCV genotype 1b.*	J05AP Antivirals for treatment of HCV infections	Metoprolol tartrate	*A beta-blocker used in the treatment of hypertension and angina, and used to reduce mortality due to myocardial infarction.*	C07AB Beta blocking agents, selective
Atazanavir	*Antiviral protease inhibitor used in combination with other antiretrovirals for the treatment of HIV.*	J05AE Protease inhibitors	Mitoxantrone	*A chemotherapeutic agent used for the treatment of secondary progressive, progressive relapsing, or worsening relapsing-remitting multiple sclerosis.*	L01DB Anthracyclines and related substances
Baricitinib	*A Janus kinase inhibitor used to treat moderate to severe rheumatoid arthritis that has responded poorly to at least one TNF antagonist.*	L04AA Selective immunosuppressants	Niclosamide	*An anthelmintic indicated in the treatment of beef, pork, fish, and dwarf tapeworm infections in adults and children.*	P02DA Salicylic acid derivatives
Bedaquiline	*Diarylquinoline antimycobacterial used in combination with other antibacterials to treat pulmonary multidrug resistant tuberculosis (MDR-TB).*	J04AK Other drugs for treatment of tuberculosis	Nilotinib	*A kinase inhibitor used for the chronic phase treatment of Chronic Myeloid Leukemia (CML) that is Philadelphia chromosome positive and for the treatment of CML that is resistant to therapy containing imatinib.*	L01EA BCR-ABL tyrosine kinase inhibitors
Bictegravir	*Bictegravir is an integrase inhibitor used to treat HIV infections.*	J05AR Antivirals for treatment of HIV infections, combinations	Nitazoxanide	*A thiazolide anti-infective used to treat infections by protozoa, helminths, anaerobic bacteria, microaerophilic bacteria, and viruses.*	P01AX Other agents against amoebiasis and other protozoal diseases
Bortezomib	*A proteasome inhibitor used to treat multiple myeloma in patients who have not been successfully treated with at least two previous therapies.*	L01XG Proteasome inhibitors	Nitrofurantoin	*An antibiotic used to treat urinary tract infections.*	J01XE Nitrofuran derivatives
Brefeldin A	*A metabolite from Penicillium brefeldianum that exhibits a wide range of antibiotic activity.*	n.a.	Ombitasvir	*A direct acting antiviral agent used in combination with other antiviral agents for the treatment of Hepatitis C Virus (HCV) infections.*	J05AP Antivirals for treatment of HCV infections
Brequinar	*Synthetic quinolinecarboxylic acid analogue with antineoplastic properties.*	n.a.	Oritavancin	*Antibacterial agent used to treat acute bacterial skin and skin structure infections caused by susceptible Gram-positive bacteria.*	J01XA Glycopeptide antibacterials
Brigatinib	*Anaplastic lymphoma kinase inhibitor used to treat anaplastic lymphoma kinase positive metastatic non-small cell lung cancer.*	L01ED Anaplastic lymphoma kinase (ALK) inhibitors	Paritaprevir	*A direct acting antiviral agent used in combination with other antiviral agents for the treatment of Hepatitis C Virus (HCV) infections.*	J05AP Antivirals for treatment of HCV infections
Capecitabine	*Nucleoside metabolic inhibitor indicated to treat colon, colorectal and breast cancer.*	L01BC Pyrimidine analogues	Pemirolast	*A medication used to treat allergies such as hay fever and allergic conjunctivitis.*	n.a.
Carbocisteine	*A expectorant mucolytic used in the relief of respiratory of COPD and other conditions associated with increased mucus viscosity.*	R05CB Mucolytics	Pibrentasvir	*Hepatitis C NS5A inhibitor used to treat Hepatitis C.*	J05AP Antivirals for treatment of HCV infections
Carfilzomib	*Proteasome inhibitor used either alone or in conjunction with a chemotherapy regimen to treat patients with relapsed or refractory multiple myeloma.*	L01XG Proteasome inhibitors	Piperacillin	*A penicillin antibiotic combined with tazobactam to treat piperacillin-resistant, piperacillin/tazobactam susceptible, β-lactamase generating strains of several bacteria.*	J01CA Penicillins with extended spectrum
Celecoxib	*An NSAID used to treat osteoarthritis, rheumatoid arthritis, acute pain, menstrual symptoms, and to reduce polyps is familial adenomatous polyposis.*	M01AH Coxibs	Ponatinib	*Kinase inhibitor used to treat patients with various types of chronic myeloid leukemia (CML).*	L01EA BCR-ABL tyrosine kinase inhibitors
Cefotiam	*A cephalosporin antibiotic used to treat a variety of bacterial infections.*	J01DC Second-generation cephalosporins	Posaconazole	*A triazole antifungal drug that is used to treat invasive infections by Candida species and Aspergillus species in severely immunocompromised patients.*	J02AC Triazole derivatives
Chloroquine	*An antimalarial drug used to treat susceptible infections with P. vivax, P. malariae, P. ovale, and P. falciparum. It is also used for second line treatment for rheumatoid arthritis.*	P01BA Aminoquinolines	Procaterol	*A beta-2 adrenergic receptor agonist and bronchodilator used for the treatment of asthma and chronic obstructive pulmonary disease (COPD).*	R03AC Selective beta-2-adrenoreceptor agonists
Clofazimine	*Riminophenazine antimycobacterial used to treat leprosy.*	J04BA Drugs for treatment of lepra	Remdesivir	*A nucleoside analog used to treat RNA virus infections including COVID-19.*	n.a.
Conivaptan	*Antidiuretic hormone inhibitor used to raise serum sodium levels.*	C03XA Vasopressin antagonists	Reserpine	*Antihypertensive and antipsychotic.*	C02AA Rauwolfia alkaloids
Darunavir	*A HIV protease inhibitor used in the treatment of human immunodeficiency virus (HIV) infection in patients with history of prior antiretroviral therapies.*	J05AE Protease inhibitors	Rifabutin	*An antibiotic used to treat mycobacterium avium complex disease in patients with HIV.*	J04AB Antibiotics
D-Mannitol	*Mannitol is a sugar alcohol used to test for asthma, to reduce intracranial and intraocular pressure, to measure glomerular filtration rate, and to manage pulmonary symptoms associated with cystic fibrosis.*	V04CX Other diagnostic agents	Rifamycin	*An antibacterial used to treat traveler’s diarrhea.*	A07AA Antibiotics
D-Sorbitol	*Laxative to relieve constipation, urologic irrigating fluid, and pharmaceutical sweetener.*	A06AD Osmotically acting laxatives	Rifapentine	*Antibiotic agent used in the treatment of pulmonary tuberculosis.*	J04AB Antibiotics
Daclatasvir	*Direct-acting antiviral agent used to treat specific hepatitis C virus (HCV) infections in combination with other antiviral agents.*	J05AP Antivirals for treatment of HCV infections	Ritonavir	*An HIV protease inhibitor used in combination with other antivirals in the treatment of HIV infection.*	J05AE Protease inhibitors
Darunavir	*HIV protease inhibitor used in the treatment of human immunodeficiency virus (HIV) infection in patients with history of prior antiretroviral therapies.*	J05AE Protease inhibitors	Roflumilast	*A selective phosphodiesterase 4 inhibitor indicated to decrease the risk of exacerbations in patients with severe chronic obstructive pulmonary disease (COPD) associated with a history of exacerbations and chronic bronchitis.*	R03DX Other systemic drugs for obstructive airway diseases
Dolutegravir	*Antiviral agent used for the treatment of HIV-1 infections in combination with other antiretroviral agents.*	J05AJ Integrase inhibitors	Saquinavir	*HIV protease inhibitor used in combination with other antiretroviral agents for the treatment of HIV-1 with advanced immunodeficiency.*	J05AE Protease inhibitors
Efavirenz	*Non-nucleoside reverse transcriptase inhibitor used to treat HIV infection or prevent the spread of HIV.*	J05AG Non-nucleoside reverse transcriptase inhibitors	Simeprevir	*Antiviral agent that inhibits HCV NS3/4A protease to treat chronic hepatitis C virus (HCV) infection in adults with HCV genotype 1 or 4.*	J05AP Antivirals for treatment of HCV infections
Elbasvir	*Antiviral and NS5A inhibitor used to treat hepatitis C infections.*	J05AP Antivirals for treatment of HCV infections	Sodium gluconate	*Gluconic acid or gluconate is used to maintain the cation-anion balance on electrolyte solutions* e.g., *fluid loss.*	n.a.
Emricasan	*A caspase inhibitor that protects liver cells from excessive apoptosis.*	n.a.	Sulfamethoxazole	*An oral sulfonamide antibiotic, given in combination with trimethoprim, used to treat a variety of infections of the urinary tract, respiratory system, and gastrointestinal tract.*	J01EC Intermediate-acting sulfonamides
Ergotamine	*A alpha-1 selective adrenergic agonist vasoconstrictor used to treat migraines with or without aura and cluster headaches.*	N02CA Ergot alkaloids	Sulfanilamide	*A sulfonamide anti-infective used to treat vulvovaginal candidiasis caused by Candida albicans.*	D06BA Sulfonamides
Flutamide	*Antiandrogen used for locally confined stage B2-C and D-2 metastatic prostate carcinoma.*	L02BB Anti-androgens	Telithromycin	*A ketolide used to treat community acquired pneumonia of mild to moderate severity.*	J01FA Macrolides
Fiboflapon sodium	*Investigated for use/treatment in inflammatory disorders (unspecified).*	n.a.	Teniposide	*A cytotoxic drug used as an adjunct for chemotherapy induction in the treatment of refractory childhood acute lymphoblastic leukemia.*	L01CB Podophyllotoxin derivatives
Fosamprenavir	*Antiretroviral agent used for the treatment and postexposure prophylaxis of human immunodeficiency virus (HIV-1) infection.*	J05AE Protease inhibitors	Tiotropium-bromide	*A long-acting bronchodilator used in the management of chronic obstructive pulmonary disease (COPD).*	R03BB Anticholinergics
Ganciclovir	*A DNA polymerase inhibitor used to treat cytomegalovirus and herpetic keratitis of the eye.*	J05AB Nucleosides and nucleotides excl. reverse transcriptase inhibitors	Tirofiban Hydrochloride	*A platelet aggregation inhibitor used to prevent thrombotic events in non-ST elevated acute coronary syndrome.*	B01AC Platelet aggregation inhibitors excl. heparin
Gemcitabine	*A nucleoside metabolic inhibitor used as adjunct therapy in the treatment of certain types of ovarian cancer, non-small cell lung carcinoma, metastatic breast cancer, and as a single agent for pancreatic cancer.*	L01BC Pyrimidine analogues	Tipranavir	*A protease inhibitor used to treat HIV-1 resistant to more than 1 protease inhibitor.*	J05AE Protease inhibitors
Glecaprevir	*A Hepatitis C NS3/4A protease inhibitor used to treat Hepatitis C.*	J05AP Antivirals for treatment of HCV infections	Tolcapone	*A catechol-O-methyltransferase (COMT) inhibitor used as adjunct therapy in the symptomatic management of idiopathic Parkinson’s disease.*	N04BX Other dopaminergic agents
Glutamine	*An amino acid commonly found as a component in total parenteral nutrition.*	A16AA Amino acids and derivatives	Valaciclovir	*An guanine nucleoside antiviral used to treat herpes exacerbations.*	J05AB Nucleosides and nucleotides excl. reverse transcriptase inhibitors
Glutathione	*For nutritional supplementation, also for treating dietary shortage or imbalance.*	V03AB Antidotes	Vancomycin	*A glycopeptide antibiotic used to treat severe but susceptible bacterial infections such as MRSA (methicillin-resistant Staphylococcus aureus) infections.*	J01XA Glycopeptide antibacterials
Grazoprevir	*An antiviral and NS3/4A protease inhibitor used to treat hepatitis C infections.*	J05AP Antivirals for treatment of HCV infections	Velpatasvir	*A NS5A inhibitor used to treat chronic hepatitis C infections in patients without cirrhosis or with compensated cirrhosis.*	J05AP Antivirals for treatment of HCV infections
Hydroxychloroquine	*An antimalarial medication used to treat uncomplicated cases of malaria and for chemoprophylaxis in specific regions. Also a disease modifying anti-rheumatic drug (DMARD) indicated for treatment of rheumatoid arthritis and lupus erythematosus.*	P01BA Aminoquinolines	Venetoclax	*A BCL-2 inhibitor used to treat chronic lymphocytic leukemia, small lymphocytic lymphoma, or acute myeloid leukemia.*	L01XX Other antineoplastic agents
Indinavir	*A protease inhibitor used to treat HIV infection.*	J05AE Protease inhibitors	Vidarabine	*An antiviral agent used to treat various viral infections.*	J05AB Nucleosides and nucleotides excl. reverse transcriptase inhibitors
Itraconazole	*An antifungal agent used for the treatment of various fungal infections in immunocompromised and non-immunocompromised patients.*	J02AC Triazole derivatives	Vismodegib	*A hedgehog pathway inhibitor used to treat patients with locally advanced or metastatic basal cell carcinoma.*	L01XJ Hedgehog pathway inhibitors

n.a.—not applicable; other substances mentioned in Kaushal et al. (2020): Aleuritic acid; CVL218 (PARP1 inhibitor); 5′Deoxy-adenosine; Dulcitol; Hexel hydrochloride; IKP-Tri-amino acid peptide; Liquiritin, (natural product); PPIases peptide; Tzaobactum; ZINC drugs; and other potential drugs, antibodies, epitopes, and ligands [19].
